# In vitro analysis of seven syphilis-causing *Treponema pallidum* strains revealed inherent growth rate differences

**DOI:** 10.1038/s41598-025-18827-9

**Published:** 2025-09-26

**Authors:** Juraj Bosák, Matěj Hrala, Petr Andrla, Eliška Vrbová, Petra Pospíšilová, David Šmajs

**Affiliations:** Department of Biology, Faculty of Medicine, Masaryk University, Kamenice 5, Brno, 625 00 Czech Republic

**Keywords:** Bacteriology, Clinical microbiology

## Abstract

Previous in vitro long-term cultivation studies of *Treponema pallidum* subsp. *pallidum* (*T. pallidum*) strains have indicated potential differences in the length of generation times among individual strains. In the present study, we have determined in vitro growth rates of seven *T. pallidum* strains including three from the Nichols-like cluster (DAL-1, Madras, and Haiti B) and four from the SS14-like cluster (Mexico A, SS14, Grady, and Philadelphia 1). Despite the observed considerable variability, the results of standard subcultures identified growth differences between the two clusters during the long-term cultivation. Furthermore, in vitro monocultures with defined inoculum revealed differences among individual strains. During three week-long binary co-cultivations of seven strains (*n* = 21), different growth rates were confirmed for individual strains (*p* < 0.001) using PCR amplicon sequencing of genomic regions differentiating treponemal-pairs. The order of strains by decreasing growth rate in vitro was DAL-1, Madras, Mexico A, Haiti B, SS14, Grady, and Philadelphia 1. While the generation time of strain DAL-1 was 32.97 h, the slowest strain, Philadelphia 1, had generation time 43.5 h. These experiments revealed significant physiological differences between the *T. pallidum* strains, which may also be involved in the variable presentation of syphilis symptoms observed in previous decades.

**Supplementary Information:**

The online version contains supplementary material available at 10.1038/s41598-025-18827-9.

## Introduction

*Treponema pallidum* subsp. *pallidum* (*T. pallidum*, TPA) is the causative agent of syphilis, a sexually transmitted disease with worldwide occurrence and increasing incidence over the past decades^1,2^. The estimated number of new cases of syphilis globally increased from 7.1 million in 2020 to 8.0 million in 2022^3^.

Based on the genomic analyses, *T. pallidum* strains are classified into Nichols-like or SS14-like cluster^4–7^. The genomic difference between clusters is less than 0.1%,^8–10^ but they differ in several parameters, including genetic diversity within each cluster^10,11^, clinical characteristics of patients (e.g., syphilis stages)^12^, and prevalence of macrolide resistance among strains^12^.

For more than a century, *T. pallidum* was considered to be continuously uncultivable under in vitro conditions^13–15^. In 2018, Edmondson et al. developed a long-term in vitro system cultivating *T. pallidum* in the presence of rabbit epithelial cells^16^. This finding started a new era of *T. pallidum* research.

During the long-term cultivation of the *T. pallidum* strains Nichols, SS14, Mexico A, UW231B, and UW249B, Edmondson et al. suggested the existence of differences in the generation times of individual strains^16–18^. Recently, Bosák et al. demonstrated that *T. pallidum* strains DAL-1 and Philadelphia 1 differ in growth rate in vivo (in rabbit experimental model) as well as in vitro showing that in vitro cultivation is a valuable tool to assess differences in *T. pallidum* growth rates^19^.

In this study, seven *T. pallidum* strains were continuously cultivated in vitro, including three strains from the Nichols-like cluster (DAL-1, Madras, and Haiti B) and four strains from the SS14-like cluster (Mexico A, SS14, Philadelphia 1, and Grady). The growth differences of the strains were analyzed during long-term in vitro cultivation and simultaneous in vitro monocultures with defined inoculum. The findings were further confirmed in a binary in vitro co-cultivation experiment.

## Methods

### Source of* T. pallidum* strains

*T. pallidum* strains DAL-1, Haiti B, Madras, Philadelphia 1, and Grady were kindly provided by Dr. D. Cox (Centers for Disease Control and Prevention, Atlanta), while SS14 and Mexico A were kindly provided by Dr. K. Hawley (University of Connecticut School of Medicine, Farmington). All *T. pallidum* strains were provided as frozen suspensions from rabbit testes with unknown concentrations of treponemal cells. For more information about used strains see Supplementary Table S1. In this study, *T. pallidum* strains were cultivated in vitro for over two years. The in vitro cultivation period of individual strains is detailed in Supplementary Figure S1.

### Long-term in vitro cultivation of ***T. pallidum*** strains

All *T. pallidum* strains were continuously cultivated in vitro using a previously published cultivation system with few modifications^16,19^. Briefly, treponemes were cultivated in a well (triplicate in a 6-well plate, 92406, Techno Plastic Products, Switzerland) containing TpCM-2 medium (4 mL) and a monolayer of rabbit cells Sf1Ep (50,000 cells). At seven-day intervals, the treponemal cultures were detached (2 × 500 µL, Trypsin-EDTA, 37 °C, 5 min) and centrifuged for depletion of Sf1Ep cells (100 × g, 5 min). The supernatant containing treponemes (5 µL aliquot) was subjected immediately to dark-field microscopy (see below) and then inoculated (typically 250 µL) to a freshly prepared well and cultivated at 34 °C in a low oxygen atmosphere (2.5%).

### Analysis of ***T. pallidum*** growth saturation under in vitro conditions

Each *T. pallidum* strain (1 × 10^6^ cells from the long-term cultures) was inoculated into seven wells and cultivated as described above for long-term cultivation, but continuously for 14 days. For each strain, one well was collected (2 × 500 µL Trypsin/EDTA) every two days, and treponemes were immediately quantified using dark-field microscopy (400× magnification, 5 µL).

Treponemal cells in 10 microscopic fields of view were counted, and the number of treponemes was calculated using the following formula: one treponeme per field equals 340,000 treponemes per mL of culture^19^.

The experiment was performed in two biological replicates.

### Quantification of ***T. pallidum*** growth in parallel in vitro monocultures

To quantify growth, in vitro cultures were adjusted to the defined treponemal inoculation dose (250,000 cells from the long-term cultures). Each biological experiment (*n* = 5) was performed in four technical replicates using a 24-well plate format (92424, Techno Plastic Products, Switzerland). Treponemes were cultivated with rabbit cells Sf1Ep (10,000 cells) in TpCM-2 medium (a final volume of 1.5 mL) under 2.5% O_2_ atmosphere. Following a seven-day cultivation period, treponemes were harvested (2 × 250 µL Trypsin/EDTA) and treponemal suspensions were immediately subjected to dark-field microscopy (400× magnification, 5 µL) as described above.

### Quantification of ***T. pallidum*** growth differences using binary in vitro co-cultivation

Seven *T. pallidum* strains were cultivated in vitro in binary co-cultivation experiment, with 21 combinations in total. Each well (*n* = 21) was inoculated with two treponemal strains (250,000 cells each, derived from long-term cultures) and cultivated for 28 days in accordance with the long-term cultivation protocol, which comprised four 7-day subcultures. The initial treponemal inoculation dose was adjusted based on dark-field microscopy, while subsequent subcultures were inoculated with a defined volume of treponemal culture (250 µl) (see above).

To quantify *T. pallidum* growth, the total DNA was isolated from two time points of each co-culture (ZymoBIOMICS™ DNA extraction kit, Zymo Research, UK) according to manufacturers´ instructions. To reduce the potential discrepancies in the viability of treponemal strains during the preparation of co-cultures (e.g., susceptibility to atmospheric oxygen), the cultures were not analyzed at day 0, but instead at day 7. The initial preparation of cultures at day 0 required a longer time than the standard subcultures.

Altogether, 13 specific genomic regions (TP0219, TP0304, TP0466, TP0488, TP0515, TP0548, TP0705, TP0733, TP0854, TP0858, TP0865, TP0966, TP0968) were selected, enabling the distinction of seven *T. pallidum* strains across 21 binary co-cultures. To ensure consistent PCR amplification efficiency, primer sites for each locus were identical in all analyzed *T. pallidum* strains, and more than one locus (2–3 different loci) per treponemal pair was amplified (for more details, see Supplementary Table S2). Furthermore, a high-fidelity polymerase and the touch-down protocol were employed under following conditions: 94 °C (1 min), 8 cycles: 98 °C (10 s), 68 – 61 °C (15 s) (every cycle has temperature lower by 1 °C), and 68 °C (6 min), followed by 35 cycles: 98 °C (10 s), 61 °C (15 s) and 68 °C (6 min), and finished by incubation at 68 °C (7 min). Each PCR reaction (25 µl) contained 5x GLX buffer (5 µl), polymerase (0.5 µl; TaKaRa PrimeSTAR^®^, Clontech, USA), water (15.3 µl), dNTPs (2 µl), primers (0.095 µl each, 100 pmol/µl), and isolated DNA (2 µl). Then, PCR products were purified (QIAquick PCR Purification Kit, QIAGEN, Germany) according to manufacturers´ instructions. Based on the amplified loci and strains in co-cultivation, PCR products were equimolarly pooled (~ 1 × 10^10^ molecules each) into 4 different mixtures for each experimental time point (i.e., D7 and D28; see Supplementary Table S2). The equimolar mixtures of PCR products were sequenced (150 bp-long paired reads, NovaSeq, Illumina, Eurofins Genomics, Germany).

Bioinformatic analysis was performed as described previously^20^. Briefly, the samples were demultiplexed to separate the data from the individual co-cultivations using Burrows-Wheeler Aligner MEM v0.7.17^21^, Samtools v1.16.1^22^, and Bedtools v2.26.0^23^. Low-quality reads were filtered out (FastQC v0.11.5 in conjunction with MultiQC v1.8)^24,25^ and adapters were trimmed (Fastp v0.20.1)^26^. The pre-processed reads were separately mapped against the whole genomes of both strains used in the co-cultivation (BWA MEM tool v0.7.17)^21^. The overall mapping quality was checked (QualiMap v2.2.2)^27^ and variants specific for individual treponemal strains were identified using Freebayes v1.3.6^28^. The number of reads specific for individual strain at both time points (i.e., D7 and D28) of the binary co-cultures was determined and the relative growth ratio for each treponemal co-culture after 21 days was calculated (i.e., fold difference between treponemal strain ratio at days 7 and 28; Supplementary Table S3). For estimation of the generation time in co-culture, the relative growth coefficient (r) was determined using the formula: r = x/21 where x = log_2_ of the relative growth ratio.

The experiment was performed in one biological replicate.

### Statistical analysis

An unpaired two-tailed Student’s t-test and linear regression were used for statistical analysis. P-values lower than 0.05 were considered statistically significant and are denoted with asterisks according to statistical significance (∗*p* < 0.05, ∗∗*p* < 0.01, and ∗∗∗*p* < 0.001). GraphPad Prism 10 software was used for calculations.

## Results

### Growth rates of ***T. pallidum*** strains during continual in vitro cultivation over two years

As of July 2024, seven *T. pallidum* strains (i.e., DAL-1, Madras, Haiti B, Mexico A, SS14, Grady, and Philadelphia 1) have been cultivated continuously for over two years using Edmondson’s in vitro system (Supplementary Figure S1). During this period, a subculture scheme employing a 20× dilution on a weekly basis produced stable cultures for Nichols-like strains (DAL-1, Madras, and Haiti B). In contrast, the SS14-like cultures (Mexico A, SS14, Grady, and Philadelphia 1) required an occasional boost with an increased inoculation dose (500–1,000 µL; i.e., a dilution of 10× and 5×) or a longer period of subculture (up to 14 days with a change of the growth medium after one week), especially strains Philadelphia 1 and Grady.

**Fig. 1:**
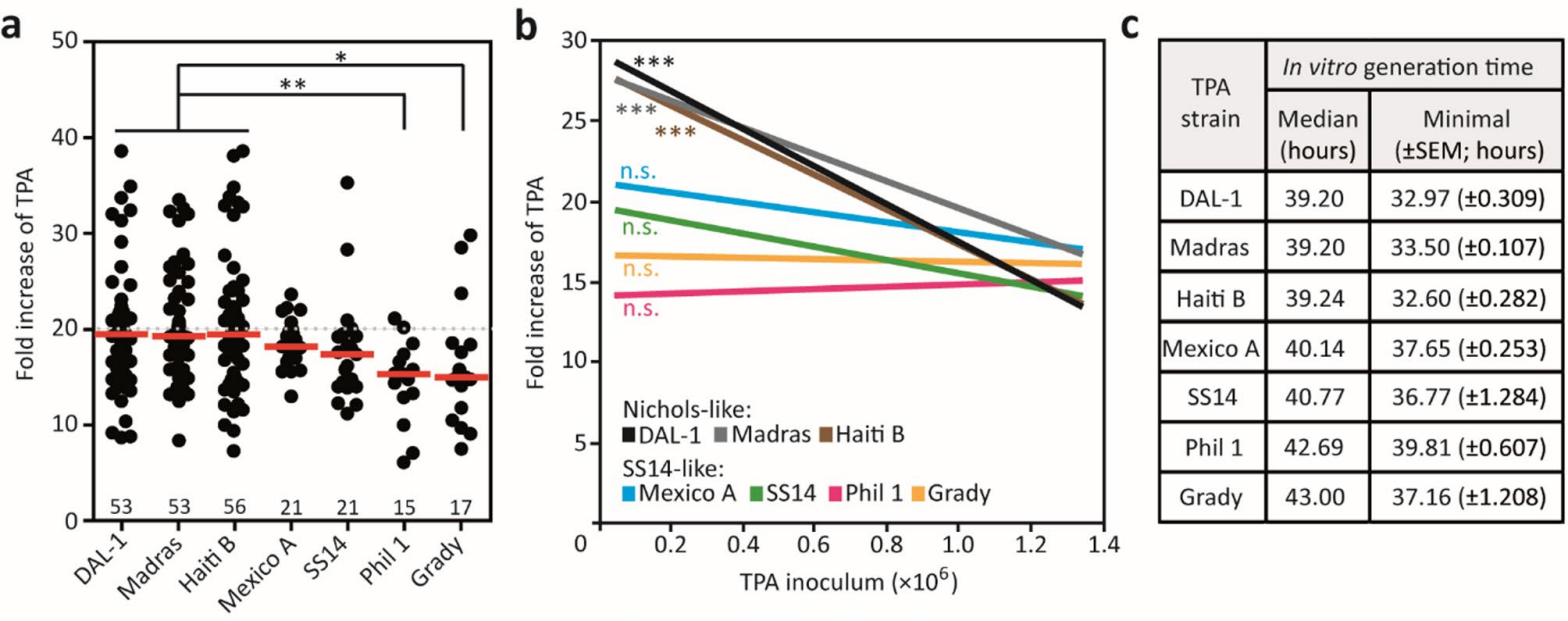
Growth rates of* T. pallidum* (TPA) strains during continual in vitro cultivation. (**a**) TPA strains differ in growth during standard 7-day long subcultures (black circles, average of TPA culture in triplicate; red bar, median). Note the wide variability of results, which likely reflects the numbers of inoculated treponemes. Unpaired Student’s t-test was used to calculate statistical significance (*p < 0.05, **p < 0.01). The number of analyzed subcultures is indicated near the x-axis. (**b**) Regression analysis revealed that the yields of SS14-like cultures were independent of the number of inoculated treponemal cells, while yields of the Nichols-like cultures were limited in high inoculation doses. Dose dependence was calculated as a non-zero slope (***p < 0.001). (**c) **Estimated in vitro generation times for individual *T. pallidum* strains. The three Nichols-like strains grew faster than SS14-like strains. The minimal generation time represents the average (± standard error) of the five lowest generation times obtained during in vitro cultivation. Please note that only standard subcultures (20× dilution every 7 days) were used for the analyses. A list of the inoculation doses and treponemal yields used in the analysis is shown in Supplementary Table S4. All subcultures of individual strains over the time are shown in Supplementary Figure S1.

The analysis of standard subcultures (20× dilution every week) revealed that the yields of Philadelphia 1 and Grady were significantly lower than those of the Nichols-like strains (*p* < 0.01 and *p* < 0.05, respectively; Fig. 1a). The inoculation doses in standard passages ranged from 6.8 × 10⁴ to 1.8 × 10⁶ of treponemal cells per culture (Supplementary Table S4). However, while growth of Nichols-like strains was dose-dependent, the yield of SS14-like strains remained constant regardless of the used inoculation dose (Fig. 1b). The observed differences in inoculum dependency support the existence of differences in *T. pallidum* growth in vitro, as all tested strains reached similar cell numbers after 14 days (32–64 × 10^6^ cells; Supplementary Figure S2).

The calculated generation time for three Nichols-like strains was 39.2 h, while generation times for individual SS14-like strains ranged between 40.1 and 43.0 h (Fig. 1c). Following Edmondson et al.,^16^ a minimal generation time eliminating the effect of suboptimal in vitro conditions in certain subcultures were also calculated for each strain. The minimal generation time for Nichols-like strains (32.6–33.5 h) was lower than for SS14-like strains (36.8–39.8 h) (Fig. 1C).

For Philadelphia 1 and Grady in vitro cultures, the tenfold dilution scheme every week (i.e., 500 µL inoculum instead of 250 µL) was found suitable for maintaining stable long-term cultures.

### Growth rate differences of ***T. pallidum*** strains in parallel in vitro monocultures

Unlike in continual cultivations, the in vitro cultures were adjusted to nearly identical cultivation conditions for all strains (i.e., the same inoculation dose, parallel cultivation in a 24-well format). Even though cultivation parameters were properly transferred from the 6-well format to the 24-well format, the yields of most treponemal cultures were lower compared to the standard 6-well format. Consequently, only two (out of five) biological experiments with the highest yield for each treponemal strain, representing eight individual wells, were further analyzed. The complete data from all five biological experiments are shown in Supplementary Table S5.

**Fig. 2:**
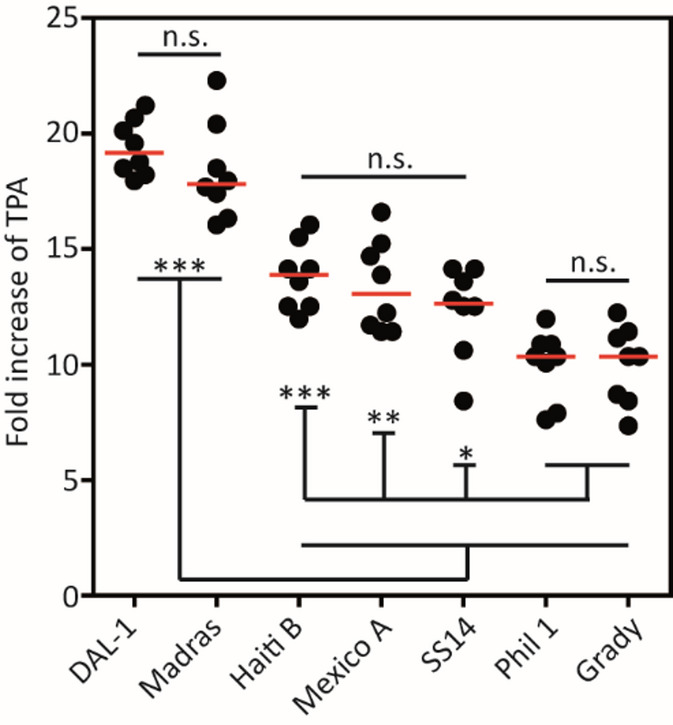
Growth rates of* T. pallidum* (TPA) strains in parallel monocultures. The growth of treponemal strains after seven days (black circles, individual wells; red bar, median) was quantified by dark-field microscopy. Data are from two biological experiments. Unpaired Student t-test was used for the calculation of statistical significance (*p < 0.05, **p < 0.01 and ***p < 0.001).

Parallel cultivation confirmed the growth differences observed between the Nichols-like and SS14-like strains, and, moreover, identified differences among individual strains (Fig. 2). Strains DAL-1 and Madras grew significantly faster than all other *T. pallidum* strains (*p* < 0.001), followed by strains Haiti B, Mexico A, and SS14; with similar growth rates. Strains Philadelphia 1 and Grady multiplied slower not only compared to Nichols-like strains (*p* < 0.001) but also compared to strains Mexico A (*p* < 0.01) and SS14 (*p* < 0.05). Interestingly, the SS14 strain grew faster than Philadelphia 1 and Grady despite the fact that all three strains differ in only over a dozen of nucleotide positions (CP004011.1, CP035104.1, and CP035193.1)^8,11^.

### Growth rate differences of ***T. pallidum*** in binary in vitro co-cultivation

**Fig. 3:**
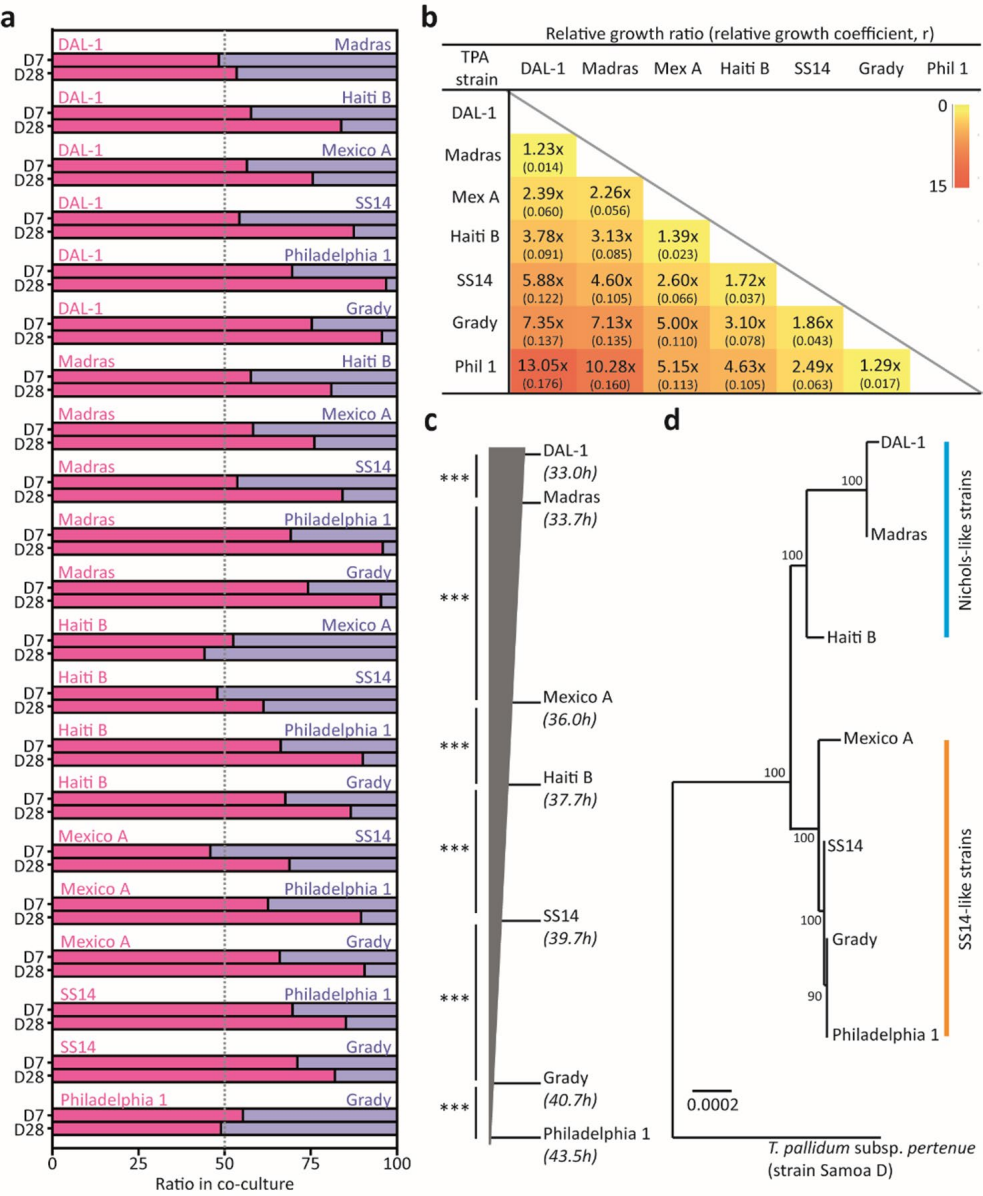
Growth rates of* T. pallidum* (TPA) strains in binary in vitro co-cultivation. (**a**) Ratios of individual strains in the co-cultivations at day 7 and day 28. The numbers of assigned reads are shown in Supplementary Table S3. (**b**) *T. pallidum* strains ordered from the fastest strain DAL-1 to the slowest strain Philadelphia 1 based on the relative growth ratio determined during 21 days (see Material and Methods). In parenthesis, the relative growth coefficient (r) shows a growth difference between two treponemal strains in generations per day. (**c**) Scheme of *T. pallidum* growth rates. The position of individual strains is derived from the treponemal pairs with the lowest relative growth ratio difference (diagonal values in panel b). The generation times were computed using the minimal generation time of the fastest strain DAL-1 (32.97 h, Fig. 1c) and the relative growth coefficient (r) for other strains (values of the first column in panel b). The statistical significance was calculated using the numbers of sequencing reads and unpaired Student t-test (***p < 0.001). (**d**) Phylogenetic analysis of the treponemal strains used in this study. Complete genomes (see Supplementary Table S1) were aligned and the evolutionary tree was inferred (Maximum likelihood and Tamura-Nei model, MEGAX v10.2.6). The scale corresponds to the number of substitutions per nucleotide. *T. pallidum* ssp. *pertenue* strain Samoa D was used as an outgroup.

To precisely determine the growth differences among seven *T. pallidum* strains in binary co-cultivations, strain-specific DNA fragments were amplified, sequenced, and the ratio between two time points 21 days apart was calculated (for more details see Methods) (Fig. 3a). In this analysis, the order of strains by their descending in vitro growth rate was determined (i.e., DAL-1, Madras, Mexico A, Haiti B, SS14, Grady, and Philadelphia 1; Fig. 3bc), and only a minimal quantitative error was found among all binary comparisons (median 7.0%). In general, the growth rates from the co-cultivation experiment are in accordance with results from our previous in vitro monocultures, which employed the long-term and parallel approaches.

Moreover, an inherent growth rate was found for each treponemal strain (*p* < 0.001) with in vitro generation times ranging from 33.0 to 43.5 h for individual strains (Fig. 3c). Interestingly, in vitro growth did not completely respect the phylogenetic classification (Fig. 3d), since SS14-like strain Mexico A overgrew the Nichols-like strain Haiti B (1.39-times over the 21-day period).

## Discussion

In this study, we have compared the growth rates of seven *T. pallidum* strains under in vitro conditions. As only a limited number of live strains were successfully isolated from patients, propagated in rabbits, and stored as viable cells^6,29–31^, our study mapped a considerable part of the available *T. pallidum* cultures.

All *T. pallidum* strains grew continuously in vitro for a period over two years, which suggests that they have been well adapted to in vitro conditions. Despite this fact, we observed differences among in vitro growth of Nichols-like and SS14-like strains. Especially, Philadelphia 1 and Grady grew significantly slower compared to other strains.

The results of standard subcultures (20× dilution every week) in long-term cultivation were highly variable, likely as a result of different numbers of inoculated treponemes during individual subcultures and/or other factors. Indeed, the fold increase of *T. pallidum* was dependent on the dose of inoculated treponemal cells, although only for faster-growing Nichol-like strains. The absence or minimal source limitation of SS14-like strains during 7-day-long subcultures likely reflects the slower in vitro growth of SS14-like strains. In fact, these strains reached the point of source limitation slightly later, between 8th and 10th day of cultivation (Supplementary Figure S2). Moreover, source limitation of SS14-like strains was observed during enrichment of subcultures (5–10× dilution every week) (see Supplementary Figure S1). Given that treponemal cells compete with eukaryotic feeder cells for nutrients, the observed differences in *T. pallidum* saturation densities may reflect the depletion of nutrients caused by the feeder cells.

For minimization of the effect of source limitation in cultivation well, we calculated the minimal generation times for each strain, which should be closer to real generation time than average/median values. The estimated generation time of 32.6–33.5 h for Nichols-like strains and 36.8–39.8 h for SS14-like strains is generally consistent with the previously reported values for strains Nichols, Mexico A, SS14, UW231B, and UW249B^16–18^. Moreover, the calculated in vitro generation times are higher but relatively close to the *T. pallidum* generation times estimated during experimental rabbit infections (~ 30 h)^19,32,33^.

Parallel cultivations of monocultures with a defined number of inoculated treponemal cells were performed to obtain more precise quantification of growth differences. Here, the significantly fastest strains DAL-1 and Madras were followed by Haiti B, Mexico A, SS14, and finally Philadelphia 1 and Grady. Identification of three subgroups of strains with different growth rates mostly agrees with the growth rates determined from the long-term cultivation and suggested that the standard subcultures were partially distorted by the presence of source limitation, most prominent among the fast-growing strains.

Despite the more standardized conditions of in vitro cultivation in comparison to animal infection^19^, the growth of *T. pallidum* in parallel wells with identical components could potentially result in inconsistencies due to the inherent complexity of growth conditions. For example, Edmondson et al. demonstrated that the growth of *T. pallidum* is influenced by the number of viable/attached rabbit cells^17^. Indeed, the results of parallel cultivation of treponemal strains revealed a relatively high extent of growth variability among experimental replicates (Supplementary Table S5). Accordingly, the in vitro co-cultivation scheme was used to avoid subtle differences in the number of rabbit cells, their age and attachment rate, and other factors that could contribute to the observed variability in parallel experiments. Furthermore, we analyzed day 7 (and day 28) of the cocultures to minimize potential differences in the viability of individual strains regarding their resistance to atmospheric oxygen during the preparation of *T. pallidum* mixtures on day 0.

Using binary co-cultivation of *T. pallidum* strains, we revealed detectable differences in the growth rates among all tested strains. The DAL-1 strain grew at the fastest rate, followed by Madras, Mexico A, Haiti B, SS14, Grady, and Philadelphia 1. In comparison to the standard continual and parallel monocultures, binary co-cultivation revealed that each strain has its own in vitro growth rate substantially differing among strains. The internal congruency in individual co-cultivations was found for all performed combinations with no evident growth antagonisms among the strains. Concurrently, the quantitative errors of co-cultivations were found higher with the increased distance from the *T. pallidum* reference comparison indicating a fair agreement of all comparisons. Moreover, the differences in the growth rates were generally consistent with the phylogenetic distance of the analyzed *T. pallidum* strains suggesting that the individual growth rates reflect genomic differences of each strain.

In general, Nichols-like strains grew faster in vitro compared to SS14-like isolates, with exception of Haiti B and Mexico A. However, both strains are somewhat exceptional. Haiti B was originally considered a *T. pallidum* ssp. *pertenue*, the causative agent of yaws, and was shown to be able to grow in hamsters^34^. Later, genomic analysis showed that Haiti B belongs to the Nichols-like cluster of syphilis-causing strains, but it differs substantially from the Nichols strain. The difference in more than 200 nucleotide positions (CP032623.1 vs. CP004010.2) represents a relatively significant divergence even for the Nichols-like strains. Mexico A harbour two recombinant loci from *T. pallidum* ssp. *pertenue* and/or *T. pallidum* ssp. *endemicum*^35^ and can be considered a rather exceptional SS14-like strain. To date, no clinical isolate related to Haiti B (allelic profile 9.83.10) has been found (PubMLST^36^. Clinical isolates related to Mexico A (allelic profile 1.13.10) were only sporadically found in North or South America^9,37^.

It is unclear whether the observed differences in growth rates reflect the in vitro growth in the presence of human cells and, more importantly, during infections of humans. However, there is currently no available in vitro cultivation system that uses human cells instead of rabbit cells. A further limitation of this study is that *T. pallidum* growth in vitro remains suboptimal, with growth rates lower than those observed in vivo in rabbits^19,32,33^. It is important to note that in vitro cultivation was not completely optimized for individual strains, and binary co-cultivation was performed in only one biological experiment. These facts could therefore affect the determined growth characteristics.

Our findings suggest that the growth of each strain represents an evolutionary selected optimum. Based on several recent epidemiological studies^6,9,10,38–42^, SS14-like strains are prevalent worldwide (80–90%); especially in European population. Moreover, frequent contemporary isolates around the world (allelic profiles 1.1.1, 1.1.3, 1.1.8, and 1.3.1)^9,37,40–43^ are closely related to Philadelphia 1 and Grady (both 1.1.1). These findings suggest that the *T. pallidum* isolates with slow growth in vitro are prevalent among the infected human population. However, further studies will be needed to discover the molecular basis of the observed differences in the *T. pallidum* growth rates.

Strains Philadelphia 1 and Grady grew considerably slower than SS14, despite the fact that all three genome sequences are highly related, with just over a dozen nucleotide differences per genome comparison. Besides four single-nucleotide polymorphisms in the intergenic regions^8^, they differ in six protein-coding genes, including TP0117 (TprC), TP0341 (MurC), TP0488 (Mcp2), TP0620 (TprI), TP0705, and TP0793, which may be involved in the observed growth differences. Nevertheless, other explanations such as occurrence of adaptive mutations in the SS14 strain cannot be excluded at this time.

Our study showed that the in vitro growth rate of *T. pallidum* strains is an inherent, individual characteristic of each strain, revealing significant growth differences across a comprehensive set of *T. pallidum* strains. These growth rate distinctions could reflect more profound physiological variations among syphilis-causing strains, potentially contributing to the variability in syphilis symptomatology that has long been observed among patients.

## Supplementary Information

Below is the link to the electronic supplementary material.

Supplementary Material 1

Supplementary Material 2

Supplementary Material 3

Supplementary Material 4

Supplementary Material 5

Supplementary Material 6

## Data Availability

All data generated and analyzed during this study are included in this published article and its supplementary files. The datasets are also available from the corresponding author on request. Sequencing data were submitted to the NCBI BioProject database under the following accession numbers PRJNA1299292.
