# Supplementary material for: In vitro analysis of seven syphilis-causing Treponema pallidum strains revealed inherent growth rate differences
Source: Sci Rep. 2025 Sep 26;15:33240. doi: 10.1038/s41598-025-18827-9 (PMC12475411; doi:10.1038/s41598-025-18827-9)
Supplement: Supplementary file 6 — Supplementary Material 6 [file 41598_2025_18827_MOESM6_ESM.pdf]

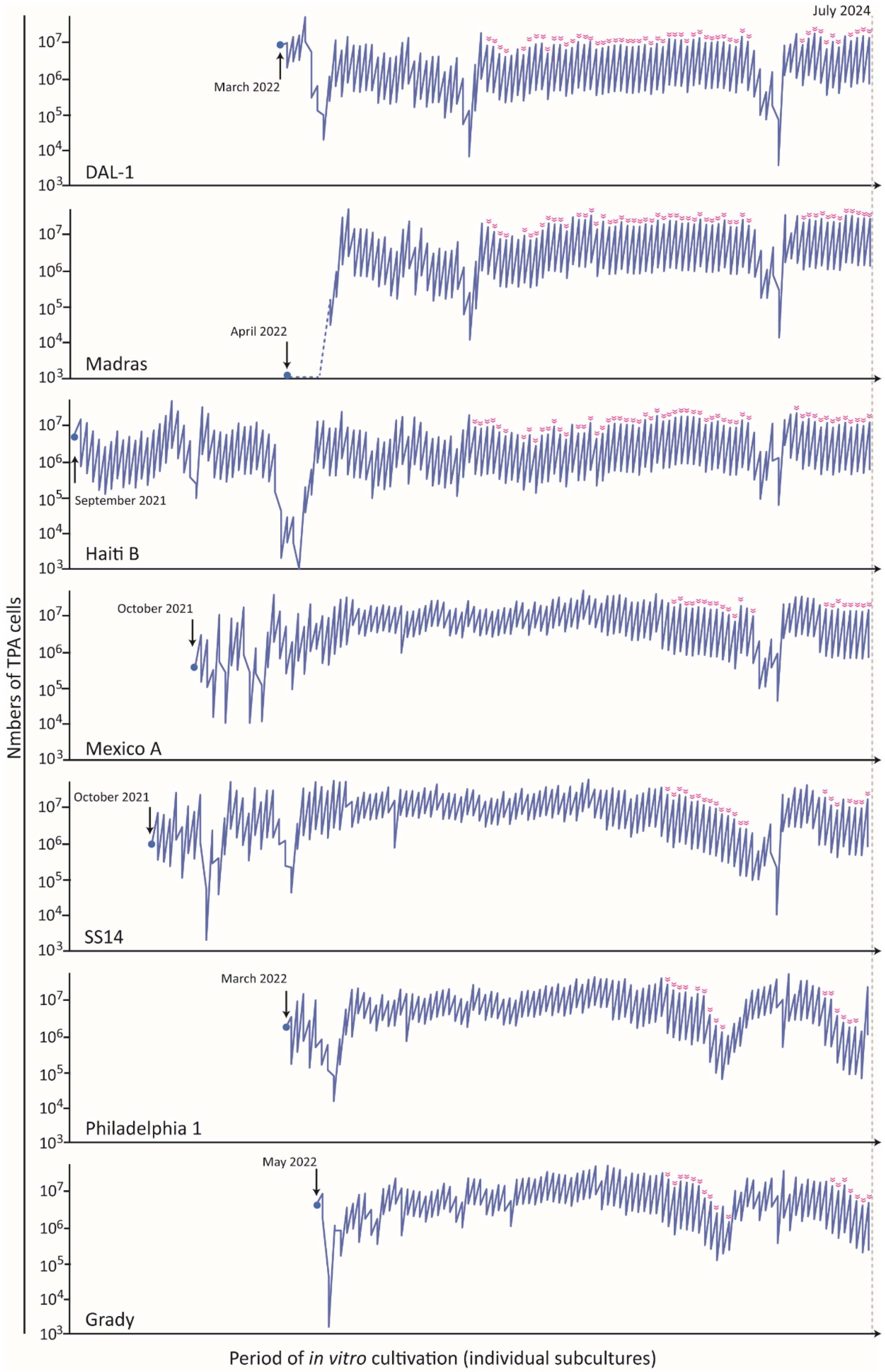


**Figure S1. Long-term in vitro cultivation of seven *T. pallidum* (TPA) strains.** This sawtooth plot shows the number of *T. pallidum* cells upon harvest and the number inoculated into subsequent subculture (mean of data from three cultivation wells per strain). The determined difference in the number of inoculated and harvested treponemes represents the *T. pallidum*

fold increase. While all strains were routinely subcultured at weekly intervals, several non-standard subcultures (up to 14 days) were also performed. For optimal presentation, the scheme does not reflect these longer, non-standard subcultures; thus, the X-axis shows only the number of passages, irrespective of their length. Pink arrows indicate standard subcultures (20× dilution every week) that were used for analysis. Notably, standard subcultures resulted in the dilution of SS14-like strains, whereas Nichols-like cultures remained stable. Concurrently, enrichment of subcultures (5–10× dilution every week) resulted in a lower fold increase for SS14-like strains (as indicated by the height of the peaks).

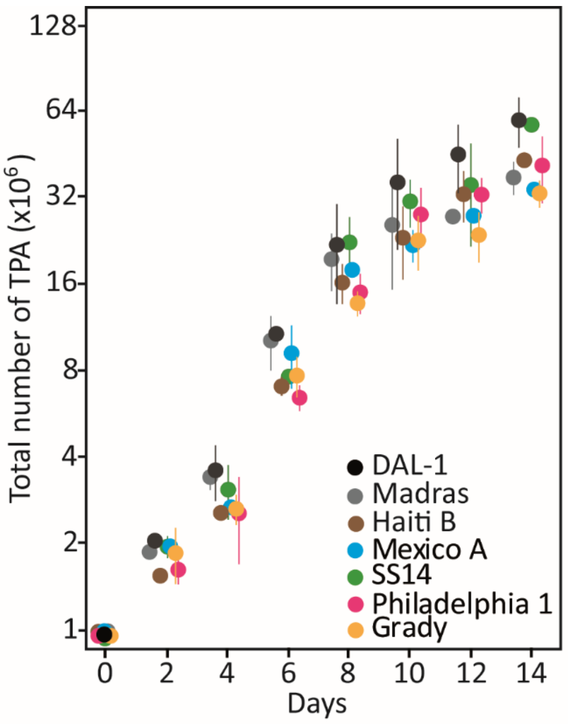


**Figure S2. *In vitro* growth of seven *T. pallidum* (TPA) strains during 14-day-long cultivation.** *T. pallidum* cultures were collected every two days and treponemes were immediately quantified using dark-field microscopy. Each dot represents an average of two biological experiments (bar, ±standard error).
